# Development and validation of a convenient formula evaluating the value and applicability of medical literature in clinical practice

**DOI:** 10.12669/pjms.306.5450

**Published:** 2014

**Authors:** Hsiao-Pei Mok, Ying Zhou, Jun-Ru Chen, Qiang Gao

**Affiliations:** 1 Hsiao-Pei Mok, West China Medical School, Sichuan University, Chengdu, Sichuan 610041, China.; 2 Ying Zhou, West China Medical School, Sichuan University, Chengdu, Sichuan 610041, China.; 3 Jun-Ru Chen, West China Medical School, Sichuan University, Chengdu, Sichuan 610041, China.; 4 Qiang Gao, Department of Cardiovascular Surgery, Guangdong General Hospital & Guangdong Cardiovascular Institute, Guangzhou, Guangdong 510100, China

**Keywords:** Evidence-based medicine, Analytic hierarchy process, Delphi method, Literature applicability, Medical literature, Evaluation method

## Abstract

***Objective:*** Evidence-based medicine offers explicit methods to evaluate the evidence grades of literature. However, evidence grades do not meet all the practical needs of physicians. This study is aimed to develop a convenient method for evaluating the clinical value of medical literature from the perspective of the clinician.

***Methods: ***A literature applicability equation was formulated through the Delphi method and the analytic hierarchy process. A consistency check was used to ascertain the efficacy of the formula. Three senior clinicians assessed 30 articles based on their clinical experiences and subjective opinions, while one independent researcher performed independent assessments of the applicability of 30 articles using the evaluation formula.

***Results:*** The literature applicability equation was Y = 3.93X_1 _+ 11.78X_2 _+ 14.83X_3 _+ 44.53X_4 _+ 24.93X_5_, where Y = literature applicability, X_1_ = years since publication, X_2_ = target question covered or not, X_3_ = sample size, X_4_ = study type, and X_5_ = journal quality. Consistency index (CI) values for the first-level indicator (“literature applicability”) and the second-level indicators (“pertinence and timeliness” and “quality of results”) were 0.0325, 0.0012, and 0.0001, respectively. The weights used to calculate the matrix indicators had satisfactory accordance (random coincidence coefficient = 0.056). A consistency check for the efficacy of the formula revealed kappa = 0.749 and *P* < .001.

***Conclusion*** *: *The developed and validated literature applicability evaluation formula may be a useful and convenient tool for identifying clinically valuable medical literature.

## INTRODUCTION

When making treatment decisions, clinicians consider not only their own experiences but also relevant studies, especially when they encounter new clinical problems. In recent decades, clinical research methods and trial registration systems have been greatly improved,^1^^,^^2^ and evidence-based medicine (EBM) has been used to classify distinct evidence levels.^3^^,^^4^ The Grading of Recommendations Assessment, Development, and Evaluation (GRADE) approach was recently developed to clarify the evidence grades of outcomes in a systematic review.^5^ However, although they offer explicit and reasonable methods to confirm the evidence strength of articles, EBM and the GRADE approach can be difficult and inconvenient for clinicians to apply in general practice. Moreover, readers are confronted with thousands of results whenever they query a literature database. For instance, Bastian et al. reported that almost 75 clinical trials and 11 systematic reviews are added to PubMed each day.^6^ Every study type has its own drawbacks that must be considered.^7^^,^^8^ An absolute conclusion can rarely be made, even for randomized controlled trials, due to the use of a poorly representative sample. Moreover, in practice, clinicians have unique and varying perspectives when assessing the value of a study. For instance, a clinician might weight studies from leading scientists more heavily, might disregard studies with high evidence grades if they are not consistent with his or her individual judgment criteria.

Sackett, one of the main initiators of EBM, stated that individual clinical expertise should be integrated with the best-available clinical evidence.^9^ However, no research to date has examined how to integrate clinicians’ experiences with the literature evidence grade. Thus, to understand the practical value of a study, it is important to consider both the evidence level of the literature and clinicians’ expertise-based internal criteria. The present study was designed to explore a concise and convenient method for assessing the applicability of literature for clinicians.

## METHODS

***Determining evaluation indicators: ***Delphi method: Evaluation indicators were determined by the Delphi method.^10^ The Delphi method is an expert panel-based forecasting method that is systematic and interactive. Multi-round questionnaires were sent to experts. After each round, the responses and reasons of each expert were summarized anonymously. In the next round, each expert was sent the summary of all experts’ answers and was given the opportunity to adjust his or her answers specifically. Finally, the ‘correct’ result was sought through consensus. We invited 12 physicians to participate in the Delphi method process. All of the participants in the Delphi method process were familiar with the fields of clinical research and epidemiology. The research group constructed the Delphi method outline and developed the questionnaire. All questionnaires were delivered by e-mail. Participants were asked to reply within 2 weeks. After every round, the research group complied the results. Final indicators were determined by at least 70% of the experts in the last round.

***Analytic hierarchy process (AHP)*** ^11^^,^^12^ ***: ***After the indicators in different levels were confirmed by the Delphi method, they were randomly listed on a form that was delivered to as many clinicians as possible, including physicians, surgeons, and anesthesiologists. Clinicians were asked to list the indicators in descending order, according to the priority that they attributed to that indicator. The results of the survey were used to calculate the weights for the indicators by AHP. After the weights were attributed, the literature evaluation formula was obtained.

***Assessing the efficacy of the literature evaluation formula: ***Three senior doctors were invited to supply one specific clinical question each and a certain number of articles that addressed their specific question. The doctors were asked to recommend a grade for each of the articles, with at least three papers for each grade. The recommendation grades were made on the basis of the clinical experiences and subjective opinions of the doctors. The grades were classified as “positive recommendation”, “general recommendation”, and “negative recommendation”. A final total of three questions and 30 articles were obtained.

One independent researcher calculated the literature score for each of the articles with the evaluation formula. The scores of the articles were sorted in a descending manner and divided into three groups: the portion of articles with the highest one-third of scores was defined as “positive recommendation,” the middle third as “general recommendation,” and the lowest third as “negative recommendation”. If the number of articles divided by 3 resulted in a remainder of 1, then one article was added to the “negative recommendation” grade; if the remainder was 2, then one article each was added to the “general recommendation” and “negative recommendation” grades.

Finally, the results with the two evaluation measures graded by the senior doctors or graded by the formula were tested by the consistency check to assess the efficacy of the evaluation formula.

***Method of blinding: ***The study design involved several levels of blinding. The experts involved in the Delphi method process did not participate in the questionnaire survey. The researcher who calculated the literature score according to the evaluation formula did not know the recommendation level made by the senior doctors, and the senior doctors who provided the articles for evaluation did not know the literature score of the articles made by the formula. Participants remained blinded until after the score had been calculated.

***Statistical analysis: ***The Delphi method and the analytical hierarchy process were used to obtain the evaluation indicators and their weights, respectively. The consistency index (CI) was calculated to test whether logical errors existed among the indicators, with CI < 0.1 indicating logical error.^11^ The accordance of the matrix of the weights of the indicators was tested by the random coincidence coefficient (CR), with CR < 0.01 indicating satisfactory accordance. The efficacy of the literature evaluation formula was assessed by consistency check; consistency was **unaccepted with kappa = 0,** was considered not well-satisfied with kappa < 0.4, and was considered satisfied with kappa ≥ 0.75.^13^ Differences with *P* < .05 were considered statistically significant.

## RESULTS

***Literature evaluation indicators: ***In the first round of the Delphi method, all of the experts confirmed the literature applicability as the first-level indictor. Three second-level indicators were identified by experts: “pertinence and timeliness,” “quality of results,” and “credibility of study”. The third-level indicators included “publication time,” “target question was covered or not,” “race or region of the participants,” “sample size,” “study type”,^14^ “journal quality,” “study performed by a professional academic organization,” and “h-index of corresponding author”.^15^ After three rounds of the Delphi method, the final indicators were determined. Table-I shows the final first-, second-, and third-level indicators that were included in this study.

***Indicator weights: ***The combination weights were calculated by the analytic hierarchy process (Table-II). The values of the indicators were assigned according to their relative clinical meaning. The indicators were individually calculated by using Arabic numbers, and the combination weights were multiplied by 100 for convenience. As a result of this process, the following formula for the literature applicability (Y) was obtained:

Y = 3.93X_1 _+ 11.78X_2 _+ 14.83X_3 _+ 44.53X_4 _+ 24.93X_5_

where X_1_ = years since publication, X_2_ = target question covered or not, X_3_ = sample size, X_4_ = study type, and X_5_ = journal quality. The CI values indicated that there were no logical errors in the determination of any of the indicator grades, and the CR value suggested that the matrix of indicator weights possessed satisfactory accordance (Table-III).

***Consistency check for the efficacy of the literature evaluation formula:*** **The** **results** **a** **re show** **n** **in** Table IV **.** Some inconsistencies in the grading were noted. One article that was graded as “positive recommendation” by a senior doctor was given a calculated score of “general recommendation” by the formula. Two articles that were graded as “general recommendation” by doctors were given calculated scores of “positive recommendation” by the formula. Two articles that were rated as “negative recommendation” by doctors were given calculated scores of “general recommendation” by the formula. Overall, for the inconsistent results, the formula tended to elevate the recommendation grade of articles compared to the grades given by the doctors. 

## DISCUSSION

In this study, we developed a method for evaluating the clinical value of literature from the perspective of the clinician. We defined the gold standard of the “real value” of articles according to the opinions of senior clinicians. In addition to improving the practicability of the results, this gold standard criterion was concise and convenient. We used the Delphi method to obtain evaluation variables and determined the weights for these variables through the analytic hierarchy process. These procedures ensured the objective and scientific nature of the literature evaluation formula. Finally, to test the validity of the method, a consistency check was used to correlate the formula with the opinions of the senior doctors (i.e., the gold standard). The results showed the satisfactory validity of the evaluation formula.

**Table-I:** Indicators of literature applicability.

**First-level indicator**	**Second-level indicator**	**Third-level indicator**	**Content of third-level indicator**
Literature Applicability	Pertinence and timeliness	Years since publication	>20 years
			>10 years and ≤20 years
			≤10 years
		Target question covered or not	No results related to target question
			Secondary results contain target question
			Major results contain target question
	Quality of results	Sample size	< appropriate sample size^a^
			= appropriate sample size
			> appropriate sample size
		Study type	Low-grade evidence b
			Moderate-grade evidence c
			High-grade evidence d
	Credibility of study	Journal quality	Regional periodical
			Medline/SCI < 3 e
			SCI ≥ 3 e

a Appropriate sample size: an estimate has been made of an effective sample size for the study.

b Low-grade evidence: 1, in vitro research, animal research; 2, expertise; 3, case series, case reports; 4, traditional review.

c Moderate-grade evidence: 1, case control study; 2, cohort study.

d High-grade evidence: 1, randomized controlled study; 2, meta-analysis or systematic review.

e 3 refers to the impact factor of SCI journals.

**Table-II:** Combination weights and value assignment of indicators.

**Third-level indicator**	**Content of third-level indicators**	**Value assignment**	**Combination weight**	**Final weight**
Years since publication (X_1_)	>20 years	1	0.0393	3.93
	>10 years and ≤20 years	2		
	≤10 years	3		
Target question covered or not (X_2_)	No results related to target question	1	0.1178	11.78
	Secondary results contain target question	2		
	Major results contain target question	3		
Sample size (X_3_)	< appropriate sample size	1	0.1483	14.83
	= appropriate sample size	2		
	> appropriate sample size	3		
Study type (X_4_)	Low-grade evidence	1	0.4453	44.53
	Moderate-grade evidence	2		
	High-grade evidence	3		
Journal quality (X_5_)	Regional periodical	1	0.2493	24.93
	Medline/SCI^a^ < 3	2		
	SCI^a^ ≥ 3	3		

a SCI: science citation index

**Table-III:** Tests of logical error and accordance of weight matrix for each indicator grade.

**Evaluation grades**		***CI *** **value**	***CR *** **value**	**Results**
First level	Literature applicability	0.0325	0.056	√^c^
Second level	Pertinence and timeliness	0.0012	- b	√^c^
	Quality of results	0.0001	- b	√^c^
	Credibility of study	-a	- b	√^c^

a The CI (consistency index) value does not need to be calculated because there is only one subindicator.

b This indicator has a second-level judgment matrix; thus, there is no need to calculate the CR (coincidence coefficient) value, because a first- or second-level judgment matrix always has complete accordance.

c The subindicators for this indicator have no logical error, and the judgment matrix has satisfactory accordance.

**Table-IV:** Consistency check for the efficacy of the literature evaluation formula.

**E** **valuation method**	**Positive recommendation**	**General recommendation**	**Negative recommendation**	**Kappa**	**P**
Clinicians’ judgment	9	11	10	0.749	< .001
Evaluation formula	9	12	9		

The clinicians prioritized “study type,” “journal quality,” “sample size,” “target question covered or not,” and “years since publication,” respectively, according to their weights. The applicability of a paper depended on the confidence of the clinician regarding the objectivity and accuracy of its results, as evidenced by the high priority attributed to the “study type”. The confidence in the results increased as the evidence strength increased from in vitro research to systemic reviews.^16^ These findings are consistent with the main idea of EBM.

Studies that are published in higher-impact journals typically require more professional and stricter peer review mechanisms for contributions. Although not all journals with high Impact Factors publish only high-quality articles,^17^ manuscripts in high-level journals are more convincing to doctors. Journals in different academic fields might have different ranges of Impact Factors. Nevertheless, for one specific literature retrieval, the search field is relatively confined. Thus, it was reasonable for “journal quality” to be chosen as an important indicator.

“Sample size” was the third-most important indicator for applicability. A study with a larger sample size might have more representative and reliable results than a smaller trial. Use of a small sample size can result in inconclusive results.^18^ For specific study types, an adequate size can be calculated by statistical methods.^18^^-^^20^ However, an appropriate sample size is only the right population. Use of a larger sample size than is necessary may result in more reliable conclusions, but more potentially confounding effects might occur during the data-collection process. These errors could, however, be reduced by applying a strict study design. Overall, it would be wise to add “appropriate sample size” as an important parameter influencing the literature applicability. And this consideration might be worthwhile for other literature evaluation systems, such as GRADE.

The factor “target question covered or not” was ranked in fourth place. This finding was somewhat inconsistent with our initial hypothesis. We had hypothesized that this indicator might be the most important, because nonrelated articles seemed useless in our initial hypothesis. This result might reflect the complexity of the clinical questions; it may be that not many eligible studies exactly covered the target questions. Clinicians have to retrieve literature that is specific for their purposes. Even among eligible studies, clinicians might hesitate to adopt the information because of discrepancies, for example, in the techniques or basic characteristics of the patients. Indirect evidence might be sufficient for clinicians to support their treatment strategies, as they prefer to obtain useful knowledge from the indirect original studies.

Finally, “years since publication” was listed as an important indicator in the formula. Clinicians were very cautious about adopting the conclusions of older articles, due to the ongoing development of techniques and therapy principles.

A consistency check was applied to test the validity of the applicability formula. The applicability grades calculated by the formula showed satisfactory consistency with the recommendation levels made by the senior doctors (defined as the gold standard in this study). After unblinding, we further investigated the reasons for differences between the recommendations by the formula and the doctors. Whereas the formula judged the quality of an article on the basis of its external characteristics, clinicians synthesized the overall information of a study, combined with their own knowledge, and then made a judgment. Thus, the judgment made by clinicians was drawn from internal information.

For example, for the “study type,” the formula gave a randomized controlled trial (RCT) or a systemic review the highest score. In contrast, clinicians might be skeptical towards the results of an RCT without detailed methods, especially if there was no evidence of the methods of randomization and allocation concealment. Clinicians were also cautious of adopting the conclusions from systemic reviews that lacked expected negative results^7^ and would downgrade such articles. These differences could explain why, compared to clinicians’ grades, the formula tended to elevate the literature grades.

Overall, the process of seeking evidence for optimizing clinical practice is full of uncertainties.^21^ This method is tightly related to clinical practice and not merely dependent on the evidence grade. The indicators in the formula are easy to obtain, and the results may be expressed in a variety of forms. For example, the formula may be displayed as an equation, or a radar chart may be made into an ‘Excel table’. By setting the formula ‘Y= 3.93X_1_+11.78X_2_+14.83X_3_+44.53X_4_+24.93X_5_’ into an Excel table and substituting for each value of X_i_, users can easily obtain the score of any article in the literature. In our department, the information secretary regularly uses this formula to filter literature. The equation is extremely convenient and easy to use. Its use does not require a researcher to read the entire article, but only enough to determine the five key factors. 

The present study offers a valid, convenient, and understandable method for evaluating literature according to its clinical relevance. Nevertheless, the sample size of this study was small, and the results require further verification.
